# An emerging targeted strategy for gastric cancer: the multi-pathway regulatory potential of Chinese medicine monomers

**DOI:** 10.3389/fonc.2026.1795543

**Published:** 2026-05-19

**Authors:** Junhui Chen, Beifen Pan, Xiaojing Cao, Yao Li, Zhiwei Tu, Chunzheng Ma, Junhui Guo

**Affiliations:** 1 The Second School of Clinical Medicine, Henan University of Traditional Chinese Medicine, Zhengzhou, Henan, China; 2 The Second Clinical College of Beijing University of Chinese Medicine, Beijing, China; 3 Spinal Surgery, The Second Affiliated Hospital of Henan University of Traditional Chinese Medicine, Zhengzhou, Henan, China; 4 Tianjin University of Traditional Chinese Medicine, Tianjin, China; 5 Hubei University of Science and Technology, Xiaogan, Hubei, China; 6 Department of Oncology, The Second Affiliated Hospital of Henan University of Traditional Chinese Medicine, Zhengzhou, Henan, China

**Keywords:** Chinese medicine monomers, gastric cancer, nanotechnology, signal pathway, traditional Chinese medicines

## Abstract

Gastric cancer (GC) ranks as the fifth most common malignancy worldwide, characterized by high incidence and poor prognosis. While current therapeutic approaches continue to evolve, their efficacy remains limited by issues such as drug resistance, adverse effects, and recurrence. Substantial evidence indicates that the pathogenesis and progression of GC are closely associated with the dysregulation of multiple key signaling pathways, including Hedgehog, Notch, PI3K/AKT, and Wnt/β-catenin. As active compounds derived from natural products, Chinese medicine monomers (CMMs) have emerged as a highly promising complementary treatment strategy due to their multi-targeting capabilities and favorable safety profiles. However, existing reviews have largely focused on single pathways or compounds, lacking a systematic analysis of multi-target synergistic regulation and the bottlenecks in clinical translation. This review systematically collates the literature published over the past 5 years (2021–2025) in CNKI, PubMed, and Web of Science. Moving beyond the previous research paradigm centered on single pathways or compounds, our aim was to systematically elucidate the molecular mechanisms by which CMMs exert their anti-GC effects through the synergistic regulation of multiple key signaling pathways. Integrated analysis demonstrates that various CMMs can effectively inhibit tumor cell proliferation, migration, and invasion by targeting these critical pathways. Their principal antitumor mechanisms involve the induction of various forms of programmed cell death—including apoptosis, autophagy, pyroptosis, and ferroptosis—while also enhancing sensitivity to conventional chemotherapy. Importantly, novel nanodelivery systems show strong potential to significantly improve the bioavailability and tumor-targeting efficiency of these monomers. In summary, CMMs offer a unique multi-targeted approach to GC treatment through synergistic pathway regulation. Future research should focus on the precise identification of molecular targets, the advancement of clinical translation, the conduct of rigorous clinical trials, and an in-depth exploration of the synergistic effects of nanotechnology-based CMMs.

## 1 Introduction

Gastric cancer (GC) is the fourth leading cause of cancer deaths and the fifth most common malignancy (1). The incidence of GC is positively correlated with advancing age. Surgical excision, radiation, chemotherapy, targeted therapy, and immunotherapy are among the current treatments for GC. However, the early diagnosis rate of GC is low, and more than 70% of patients are diagnosed at an advanced stage. The overall prognosis remains unsatisfactory even after the primary lesion has been surgically removed (2). With the development of tumor molecular science, researchers continue to discover new tumor targets. Tumor targets have a far higher therapeutic potential than traditional GC therapies such as chemotherapy and radiotherapy and thus have become a hotspot for GC drug research. Although conventional therapies, including surgery and radiotherapy, have improved clinical outcomes, the limitations of single-target treatments are no longer able to satisfy the complex medical needs of patients with GC (3). Therefore, it is necessary to develop more effective or adjuvant therapeutic or preventive therapies for GC.

Chinese medicine monomers (CMMs) have gained significant attention in cancer prevention and treatment due to their multi-component nature, multi-target mechanisms, and favorable toxicity profiles. By controlling signaling pathways such as PI3K/AKT and JAK/STAT, CMMs can prevent GC cells from proliferating and spreading, as well as trigger programmed cell death such as apoptosis and ferroptosis (4–6). For example, dihydroartemisinin (DHA) inhibits glutathione peroxidase 4 (GPX4) to induce ferroptosis in GC cells (7), and baicalin induces GC cell pyroptosis by activating the NF-κB–NLRP3 axis (6). In addition, the combination of herbal medicines with a nanodelivery system significantly enhanced the bioavailability and targeting of hydrophobic components such as curcumin (Cur) and resveratrol (Res) (8, 9). These studies on individual components and their *in vitro* mechanisms of action have laid a crucial foundation for elucidating the anti-GC activity of CMMs. Building on this foundation, further systematic integration and analysis from the perspectives of multi-pathway synergistic regulation and clinical application potential will contribute to a more comprehensive understanding of their therapeutic value.

**Figure 1:**
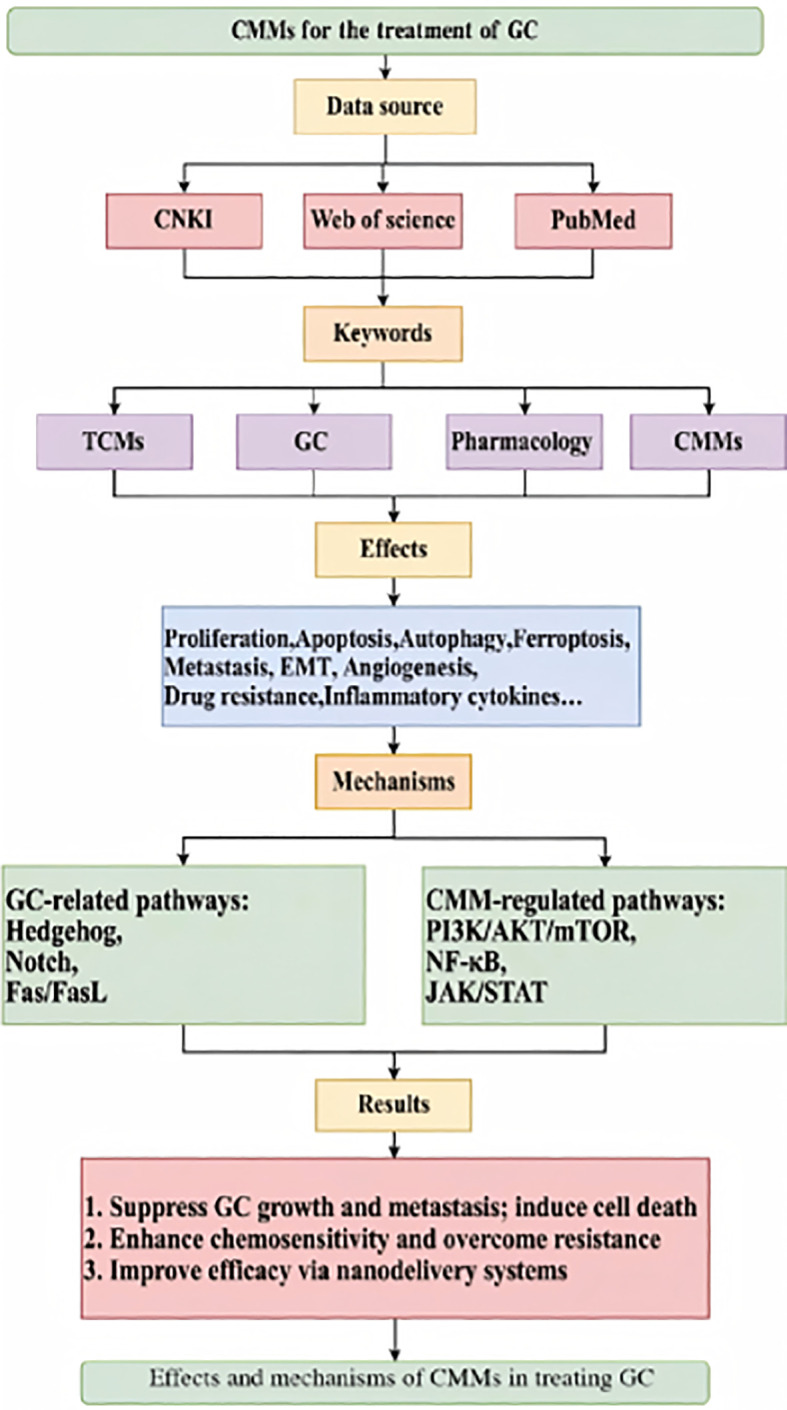
Diagram of the research process.

This review aimed to synthesize basic and selected clinical research from the past 5 years to systematically elucidate the molecular mechanisms of the core signaling pathways in GC, with a particular focus on the mechanisms by which CMMs treat GC by interfering with these signaling pathways. It also provides a critical summary of the field, assessing it in terms of the strength of evidence, consistency of mechanisms, potential biases, and the feasibility of clinical translation. Furthermore, this review will explore methods of using nanotechnology to overcome the pharmacokinetic limitations of traditional Chinese medicine (TCM), thereby establishing a scientific basis for the development of CMM-based combination therapies for GC. The research flowchart is shown in **Figure 1**.

## 2 Main signaling pathways in GC

GC is closely related to the abnormal activation or inactivation of multiple signaling pathways that are involved in tumor progression through the regulation of cell proliferation, apoptosis metabolism, immune escape, and other processes. The following systematic description of the molecular mechanisms of the key signaling pathways in GC and their potential targeting value in therapy lays the foundation for the treatment of GC by CMMs by intervening in these pathways.

### 2.1 Hedgehog signaling pathway

Through the regulation of cell proliferation and differentiation, the highly conserved Hedgehog (Hh) signaling pathway is essential to embryonic development. This pathway was first identified in *Drosophila melanogaster*, and its aberrant activation is closely related to the development of GC (10). This signaling pathway includes important target genes, including *PTCH1/2* and *GLI1*, as well as transcription factor components such as Sonic Hh (SHh), Indian Hh (IHh), Desert Hh (DHh), and Smoothened (Smo) (11). In GC, aberrant activation of the Hh pathway (e.g., ligand-dependent autocrine/paracrine or gene mutations) is closely associated with tumorigenesis and has become a potential therapeutic target (11).

The Hh ligand binds to *PTCH1*/*2* and derepresses the Smo protein, which in turn activates transcription factors such as *GLI1* (11). *GLI1* acts as a key downstream effector molecule, which promotes the proliferation and metastasis of GC cells through the upregulation of target genes such as *INHBA*. *Helicobacter pylori* infection can upregulate *GLI1* expression through *N*^6^-methyladenosine modification and exacerbate GC progression (12). ADP-ribosylation factor 6 knockdown combined with Hh pathway inhibitor synergistically inhibited GC cell proliferation and migration (12). Single-cell sequencing further revealed that a subpopulation of cyclin D1+ fibroblasts in the GC microenvironment is highly enriched for Hh pathway activity, which may impair the effects of immunotherapy and chemotherapy by paracrine Hh ligands (13).

In summary, the multidimensional regulatory mechanisms of the Hh pathway in GC provide a crucial foundation for the development of targeted treatment strategies. Existing research has largely focused on the TCM formula “Sancao Diaowei decoction,” which inhibits Hh pathway activity by downregulating SHh and *GLI1* and upregulating *PTCH1*, thereby significantly delaying tumor progression in a rat model of GC (14). The synthetic drug apatinib inhibits GC stem cells by suppressing the SHh pathway, thereby inhibiting the development of GC (15). However, direct evidence regarding the Hh pathway in GC remains relatively limited, suggesting that this axis represents a research area worthy of priority attention in the future. Prior to clinical application, it is necessary to further validate the selectivity of pathway inhibitors and to clarify which patients, based on molecular subtypes or microenvironmental characteristics, are more likely to benefit from Hh-targeted interventions.

### 2.2 Notch signaling pathway

The Notch signaling pathway is an evolutionarily conserved signaling pathway involved in the maintenance of tissue homeostasis by regulating cell proliferation, differentiation, and apoptosis and determining cell fate (16). This pathway consists of one transmembrane receptor (Notch1–4) and five ligands (DLL1/3/4 and JAG1/2) (17). Its activation is dependent on ligand–receptor binding between neighboring cells, which ultimately regulates the transcription of target genes through the release of Notch intracellular structural domains (NICDs) (16). The primary manifestation of aberrant Notch pathway activation in GC is an oncogenic impact, and tumor growth and a poor prognosis are closely linked to its dysregulation.

Clinical studies have shown that the components of the Notch pathway are significantly overexpressed in GC tissues, with the overexpression of DLL1/3/4 and JAG2 correlating with shorter overall patient survival (17). Notch1 may promote GC lymph node metastasis through the PTEN–ERK1/2 signaling pathway (18), whereas Notch3 may activate the AKT pathway through the upregulation of *PHLDB* expression to drive GC occurrence (19). Platelet-responsive protein 2 has been demonstrated to preserve the traits of cancer stem cells via Notch3 (20), and glutamate-rich WD repeat sequence containing 1 enhances the expression of ADAM metallopeptidase structural domain 17 (21), which in turn promotes the release of NICDs and the activation of the Notch signaling pathway. GC invasion is accelerated as a result (21). In addition, the Notch pathway interacts significantly with the tumor microenvironment (TME): Ubiquilin-4 inhibits antitumor immunity by upregulating programmed death-1 expression in tumor cells through the activation of the Notch signaling pathway (22), whereas fibrinogen-like protein 1 promotes immune escape by activating the Notch signaling pathway to inhibit CD8 T-cell activation (23).

Targeting the different components of the Notch pathway exhibits therapeutic potential. For example, miR-491-5p and miR-875-5p negatively regulate Notch3 to exert oncogenic effects (19). Notch4 mutations have been associated with enhanced tumor immunogenicity and inflammatory antitumor immunity, and Notch4 mutations may serve as a predictive marker for the efficacy of immune checkpoint inhibitor (ICI) therapy for the treatment of cancer, including GC (24).

However, further research is still required into the function of the Notch pathway and its dependence on the microenvironment, as its biological effects may be jointly influenced by the receptor subtypes, mutation status, and the immune context. From the perspective of CMM-targeted therapy, the Notch pathway is closely associated with cancer stemness, immune evasion, and ICI efficacy; however, evidence for direct monotherapy intervention in GC remains limited at present. Future efforts could focus on the screening of CMMs capable of inhibiting NICD release, Notch3/4 activation, or Notch-associated immunosuppressive phenotypes and the exploration of their synergistic effects with existing therapies (such as immunotherapy and chemotherapy).

### 2.3 PI3K/AKT signaling pathway

The PI3K/AKT pathway is a critical signaling system that controls cell survival, metabolism, and proliferation, and abnormal activation is linked to the development of GC (25). This pathway consists of components such as the catalytic subunit (p110), the bridging/regulatory subunit (p85), phosphatidylinositol-3,4,5-triphosphate (PIP3), and the downstream effector molecule AKT (26). Upon binding of the ligand to the receptor, PI3K catalyzes the phosphorylation of phosphatidylinositol-4,5-bisphosphate (PIP2) to PIP3. In addition, PIP3 is also a docking site for phosphatidylinositol 3-phosphate-dependent kinase (PDK1) and the mammalian target of rapamycin (mTOR) complex 2 (27), which promotes the phosphorylation of the threonine 308 site (Thr308) of AKT proteins by PDK1 and the activation of the serine 473 site (Ser473) of AKT proteins through mTOR complex 2, leading to the full activation of AKT (28). AKT phosphorylates the pro-apoptotic factor BAD to elevate Bcl-2 and caspase-9 in order to inhibit apoptosis (29) while activating pathways such as mTOR complex 1 to promote tumor growth (30).

The PI3K/AKT pathway mediates the bidirectional action of promotion and repression in GC through a multi-gene regulatory network. It was found that overexpression of the transcription factor MYB proto-oncogene-like 2 promotes GC cell proliferation and inhibits apoptosis through the activation of the PI3K/AKT and BCL-2/BAX/cleaved caspase-3 signaling pathways, and the cell cycle protein B2 (CCNB2)–rhomboid-like 2 protein (RHBDL2)–PI3K/AKT axis induces cellular epithelial–mesenchymal transition (EMT) in cells to promote GC progression (31, 32). However, metallothionein 1G reverses the malignant phenotype of GC by blocking this signaling pathway (33). Furthermore, this pathway is closely associated with chemotherapy resistance: the Forkhead box A1 (FOXA1)/meiotic nuclear division 1 homolog (MND1)/transketolase (TKT) axis promotes the progression of GC via the PI3K/AKT signaling pathway, while simultaneously reducing sensitivity to platinum-based chemotherapy (34).

CMMs also exhibit regulatory potential: flavonoids (e.g., iridin and catechin) inhibit GC cell proliferation and migration while promoting GC cell apoptosis by inhibiting the PI3K/AKT pathway (35, 36). Pachymic acid (PA) blocks PI3K/AKT signaling by targeting PIK3 regulatory subunit 1, inhibits the cell cycle, and promotes GC ferroptosis (37, 38).

In summary, small-molecule inhibitors and natural compounds targeting the PI3K/AKT pathway have demonstrated therapeutic potential. It is worth noting that the PI3K/AKT pathway is currently one of the therapeutic targets with the most robust evidence for CMMs: flavonoids (e.g., iridins and catechins), terpenoids (e.g., PA, bufotalin, DHA, and triptolepine), and others can exert effects such as inhibiting proliferation, inducing apoptosis/ferroptosis, suppressing migration, and reversing chemotherapy resistance by inhibiting the PI3K/AKT pathway or its upstream/downstream nodes. This will be detailed later in this paper. Combining these with chemotherapy or immunotherapy is expected to enhance therapeutic efficacy, and this is anticipated to become a forward-looking treatment strategy for GC.

### 2.4 Fas/FasL signaling pathway

The Fas/FasL signaling pathway is a key regulatory pathway for apoptosis in tumor cells, consisting of the death receptor Fas (CD95/APO-1), the ligand for FasL (CD95L/APO-1L), the bridging protein FADD (Fas-associated death domain), the initiating caspases (8/10), and the effector caspases (3/6/7), and mediates exogenous apoptosis through the death-inducible signaling complex (DISC) (39, 40). The binding of Fas to its ligand FasL triggers an exogenous apoptotic cascade, which plays an important role in the maintenance of tissue homeostasis and the clearance of abnormal cells (41, 42). Upon ligand binding, the Fas trimer recruits the FADD protein, and FADD and pro-caspase 8 are recruited to form the DISC, while pro-caspase 8 undergoes self-cleavage and is converted to activated caspase 8, which activates a large number of caspases, including downstream effectors such as caspase 3. This results in a protease cascade that ultimately leads to the apoptosis of tumor cells (39, 40).

However, in GC, the dual roles of the Fas/FasL pathway—both pro-tumor and antitumor—complicate its mechanism: within tumor cells, Fas activation induces apoptosis, thereby exerting an antitumor effect; however, in the TME, FasL, which is highly expressed by tumor cells, induces apoptosis in infiltrating lymphocytes, thereby promoting immune evasion and tumor progression (43). Tumor infiltration with lymphocyte apoptosis is a relevant mechanism for immunotherapy resistance, which can be blocked by interfering with the Fas/FasL pathway (44). Liu et al. found that leucine-rich repetitive neuron protein-1 was significantly upregulated in GC, which reduces apoptosis through the inhibition of the Fas/FasL pathway and is correlated with poor patient prognosis (45). In addition, celecoxib induces apoptosis and inhibits the growth of GC cells by regulating the protein expression of Fas/FasL, as well as Bcl-2 (46). Notably, certain CMMs exert their therapeutic effects by modulating the Fas pathway: iridin activates Fas-mediated exogenous apoptosis through the inhibition of PI3K/AKT signaling while inducing G2/M-phase GC cell cycle arrest (35).

Targeting the Fas/FasL pathway requires addressing its microenvironmental dependence and dual regulation, as well as its interaction with chemoresistance and ICIs. The existing evidence on CMMs suggests that some CMMs may enhance cell death by synergistically activating Fas-mediated extrinsic apoptosis through the inhibition of the PI3K/AKT pathway or by upregulating the Fas/FasL-related apoptotic signaling. Consequently, this pathway is better suited for discussion as a “synergistic pro-apoptotic target” rather than as an isolated target. Future research should focus on the development of small molecules and CMMs capable of selectively activating pro-apoptotic signals and the exploration of their synergistic effects with immunotherapy in order to overcome the bottlenecks in clinical translation.

### 2.5 Wnt/β-catenin signaling pathway

The Wnt/β-catenin signaling pathway is a key pathway that regulates cell proliferation, differentiation, and stem cell maintenance. In gastric tissue, it is involved in both normal epithelial renewal and the maintenance of homeostasis, but may also promote the development and progression of GC when abnormally and persistently activated. The components of this pathway include the Wnt ligand, Frizzled receptor, LRP (low-density lipoprotein receptor-associated protein), and the downstream effector molecule β-catenin (47). In normal cells, a destruction complex consisting of the adenomatous polyposis coli (APC) oncogene, axis inhibition protein, glycogen synthase kinase 3, and casein kinase 1 maintains β-catenin signaling at low levels. When cells are exposed to Wnt ligands, the activity of this destruction complex is transiently inhibited, and β-catenin undergoes nuclear translocation and interacts with the transcription factors in the nucleus, leading to the transcription of Wnt target genes and promoting tumor progression (48).

Overexpression of the Wnt/β-catenin signaling pathway is significantly associated with the invasiveness, metastasis, and chemoresistance of GC. Studies have indicated that this pathway is also associated with chemoresistance in GC and constitutes a key signaling pathway underlying the development of resistance to trastuzumab in GC cells (49). Research suggests that neuropilin-1 may promote GC proliferation and inhibit autophagy in GC cells by activating the Wnt/β-catenin signaling pathway (50). On the other hand, the β-catenin/TCF4 transcriptional complex directly binds to and induces the expression of GPX4 in its promoter region, which inhibits ferroptosis and enhances GC tolerance to cisplatin, and TCF4 deficiency or Wnt signaling inhibition may induce ferroptosis *in vivo* and promote cisplatin sensitivity (51). In addition, the hyperactivation of this pathway contributes to the maintenance of GC cell stemness and can promote GC relapse and enhance drug resistance (52).

Targeting the Wnt/β-catenin pathway is an important strategy to improve treatment in GC. A Chinese herbal compound (Banxia Xiexin decoction) inhibited GC proliferation and induced apoptosis by downregulating the Wnt/β-catenin signaling pathway (53). In addition, a plant-based medicinal food has anti-GC effects by reversing EMT transformation, inducing apoptosis, and inhibiting GC proliferation by blocking the Wnt/β-catenin signaling pathway (54). However, the role of the Wnt/β-catenin pathway in GC is not merely oncogenic: this duality is manifested in the fact that, while it is indispensable for embryonic development and the maintenance of normal tissue homeostasis (physiological function), its abnormal activation in GC drives tumor progression, stem cell maintenance, and chemoresistance (pathological function). Future research should focus on elucidating the functional status and therapeutic windows of Wnt signaling in different patient subgroups, developing subtype-selective inhibitors, unraveling the mechanisms of resistance, and devising combination therapy strategies based on patient molecular subtyping to optimize therapeutic efficacy.

### 2.6 Others

In addition to the above core pathways, the roles of signaling axes such as mitogen-activated protein kinase (MAPK/MEK) and JAK/STAT in the development of GC are gradually gaining attention. These pathways provide new directions for targeted therapies by regulating biological processes such as EMT, apoptosis, autophagy, and chemoresistance.

The MAPK pathway has a multidimensional regulatory role in GC, which consists of four components, namely, RAS (rat sarcoma viral oncogene homolog), RAF (rapidly accelerated fibrosarcoma), MEK, and ERK (extracellular signal-regulated kinase), specifically including the important components of ERK5, ERK, JNK, and p38/MAPK (55). Studies have shown that raddeanin A induces apoptosis and autophagy in GC cells through the activation of the tyrosine phosphorylated protein kinase (p38MAPK) signaling pathway (56), and aberrant MAPK pathway activation has also been shown to be closely associated with chemoresistance. For example, JunB proto-oncogene promotes oxaliplatin resistance in GC cells through the MAPK signaling pathway (57).

Aberrant activation of the JAK/STAT pathway also plays a critical role in GC. The pathway consists of three main components: tyrosine kinase-related receptors, the JAK family (JAK-I, JAK-II, JAK-III, and TYK-II), and the STAT family (STAT-I, STAT-II, STAT-III, STAT-IV, STAT-Va, STAT-Vb, and STAT-VI) (58). Persistent activation of STAT3 can epigenetically silence miR-193a, upregulate its downstream target YWHAZ, and ultimately promote tumor metastasis (59). Notably, inhibition of the JAK2/STAT3 pathway significantly enhanced the sensitivity of GC cells to 5-fluorouracil (5-FU) (60), suggesting its potential value in combination with chemotherapy.

Taken together, the MAPK and JAK/STAT pathways are involved in GC progression by regulating the malignant phenotype and microenvironmental interactions of tumor cells. Targeted inhibitors or CMMs acting on these pathways have demonstrated therapeutic potential: Cur and berberine inhibit JAK/STAT3 signaling to reduce tumor cell survival and chemotherapy resistance; triptolepine also inhibits JAK2/STAT3. On the other hand, raddeanin A, crocin, bufotalin, and andrographolide derivatives can influence apoptosis, EMT, angiogenesis, and drug sensitivity via MAPK/ERK/p38/JNK-related pathways, as will be detailed later in this paper. However, their clinical translation requires further exploration of the subtype selectivity, resistance mechanisms, and synergistic effects with other therapies. The main signaling pathways of GC are shown in **Table 1**.

**Table 1:** Main signaling pathways of gastric cancer (GC).

Signaling pathway	Composition	Function	Reference
Hh signaling pathway	SHh, IHh, DHh, Smo, PTCH1/2, and GLI1, among others.	Inhibit the Hh pathway, which can delay the development of GC	(10–15)
Notch signaling pathway	Notch1–4, DLL1/3/4, and JAG1/2, among others	Mainly promote the development of GC	(16–24)
PI3K/AKT signaling pathway	P110, P85, PIP3, and AKT, among others	Promote the development of GC	(25–38)
Fas/FasL signaling pathway	Fas/FasL, FADD, caspase (8/10), and caspase (3/6/7)	Dual action: it can induce apoptosis in tumor cells, but can also mediate immune evasion.	(35, 39–46)
Wnt/β-catenin signaling pathway	Wnt, Frizzled, LRP, and β-catenin, among others	In the majority of studies, promoting the occurrence of GC	(47–54)
MAPK signaling pathway	RAS, RAF, MEK, and ERK	Promote the development of GC	(55–57)
JAK/STAT signaling pathway	Tyrosine kinase-related receptors, the JAK family, and the STAT family	Promote the development of GC	(58–60)

Overall, the current CMMs exhibit multidimensional characteristics in their therapeutic targeting of the GC signaling pathways: they not only directly inhibit kinases and transcription factors in multiple proliferation and metabolic pathways—such as PI3K/AKT, Wnt/β-catenin, MAPK, and JAK/STAT—but also demonstrate potential value in modulating specific microenvironmental pathways. This synergistic, multi-target, and multi-pathway targeted regulation is precisely the core advantage that makes CMMs promising in overcoming the drug resistance associated with traditional single-target synthetic drugs.

## 3 CMMs and their effects on signaling pathways

CMMs exert multidimensional antitumor effects by regulating GC-related signaling pathways, and their mechanisms include inhibition of proliferation, induction of programmed death, reversal of drug resistance, and synergistic enhancement of therapeutic efficacy by nanodelivery systems. The mechanism of action and experimental basis of the representative CMMs are summarized below.

### 3.1 Flavonoids

#### 3.1.1 Baicalin

Baicalin, a flavonoid extracted from the root of *Scutellaria baicalensis*, family Labiatae, possesses anti-inflammatory, antioxidant, and antitumor activities. Studies have shown that baicalin inhibits GC cell proliferation and induces programmed cell death through multi-pathway regulation. Its mechanisms include: 1) mitochondrial apoptotic pathway: upregulation of Bax expression, downregulation of Bcl-2 expression, and activation of caspase-3 and caspase-9 expression, inhibiting GC proliferation and inducing endogenous apoptosis (61); 2) ferroptosis: promotion of reactive oxygen species (ROS)-mediated ferroptosis, enhancing the chemosensitivity of oxaliplatin-resistant GC cells (5); and 3) cell pyroptosis: activation of the NF-κB/NLRP3 signaling axis, inducing pyroptosis (6). In addition, baicalin synergistically enhances the efficacy of 5-FU, which exacerbates lipid peroxidation and further promotes ferroptosis through a ROS-dependent mechanism (62). The aforementioned multi-targeted effects suggest that baicalin may act as a CMM to augment chemotherapy by inhibiting the progression of GC through the synergistic regulation of apoptosis, ferroptosis, and pyroptosis pathways.

#### 3.1.2 Silymarin

Silymarin (silybin) is a flavonoid lignan compound extracted from the seed coat of *Silybum marianum*, a medicinal plant of the Asteraceae family with antioxidant, anti-fibrotic, anti-inflammatory, and immunomodulatory properties. Its anti-GC effects are manifested as follows:

1. Anti-inflammatory effects: Silymarin alleviates *H. pylori*-induced gastric mucosal inflammation by inhibiting the NF-κB/STAT3 signaling axis and downregulating the expression of COX-2 and inducible nitric oxide synthase (iNOS) (63). Studies have shown that chronic inflammation caused by dysbiosis is a key driver mediating the remodeling of epigenetic abnormalities, such as DNA methylation, in gastric precancerous lesions (64). Based on this mechanism, silymarin holds promise in the prevention of *H. pylori*-induced gastritis and its subsequent epigenetic changes and the development of GC.
2. Optimization of therapeutic efficacy via nanodelivery systems: Silymarin-loaded solid lipid nanoparticles (SLNs) not only improve its pharmacokinetic properties but also demonstrate synergistic antitumor effects. By downregulating miR-181a and upregulating miR-34a, they inhibit the TGF-β/SMAD3/β-catenin pathway; simultaneously, they antagonize the expression of anti-migratory genes by E-calcimycin, thereby blocking the proliferation and migration of GC cells and inducing apoptosis (65). The above study suggests that silymarin may enhance bioavailability via a nanodelivery system and play a multi-targeted interventional role in the adjuvant treatment of *H. pylori*-associated GC.

### 3.2 Polyphenols

#### 3.2.1 Resveratrol

Resveratrol (Res) is a naturally occurring polyphenolic compound described and isolated from *Veratrum grandiflorum*, and its anti-GC effects are mainly realized through the following mechanisms:

1. Inhibition of GC cell proliferation, invasion, and metastasis, as well as induction of apoptosis: Res regulates the malignant biological behavior of GC through multiple targets. Studies have shown that Res can reduce acetyl heparinase activity in GC cells and inhibit tumor invasion and metastasis, with the mechanism related to the increase of superoxide dismutase (SOD) activity and the inhibition of NF-κB transcriptional activity by Res (66). Another study found that Res could inhibit proliferation, migration, and invasion and induce apoptosis in human GC cells by regulating the MALAT1/miR-383-5p/DDIT4 pathway (67). In addition, network pharmacological analysis further confirmed that Res inhibited the GC cell cycle and induced apoptosis by targeting FOS (FBJ murine osteosarcoma viral oncogene homolog) and MMP-9 (matrix metallopeptidase-9) (68).
2. Enhancement of chemosensitivity: Res can reverse chemoresistance by modulating the tumor metabolism and stem cell properties. It has been found that 4′-bromo-Res (4-BR), a Res analog, inhibits the SIRT3/c-Jun N-terminal kinase pathway to reduce GC cell stemness and increase chemosensitivity, which is beneficial for GC stem cell targeted therapy (69). Another study showed that the combination of Res and 5-FU synergistically activated the apoptosis and autophagy pathways, significantly enhanced the efficacy of 5-FU on GCs, and attenuated cardiotoxicity (70).
3. Nanodelivery system to optimize efficacy: the low water solubility and bioavailability of Res limit its clinical application, and even the accumulation of high doses of Res in tumor tissues may impair its efficacy (71). To address this challenge, studies have constructed pH-responsive nanocarriers, e.g., Res@ZIF-90, which inhibit the proliferation and migration of HGC-27 cells by disrupting mitochondrial homeostasis (e.g., promoting the expression of mitochondrial fission-associated proteins and inhibiting mitochondrial fusion-associated proteins) in GC cells (9). Another study utilized mesoporous silica nanoparticles loaded with Res (MSN-Res) and found that its antitumor activity was superior to that of free Res both *in vivo* and *in vitro*, suggesting the potential for the application of nanodelivery systems (72).

In conclusion, Res inhibits GC progression through multiple pathways, and its combination with chemotherapy or nanotechnology can further optimize the efficacy, making it a promising natural anti-GC candidate. However, further work is required to address issues such as the long-term storage stability of the drug delivery system, the consistency of batch production, and the potential toxicity of the nanomaterials.

#### 3.2.2 Curcumin

Curcumin (Cur) is a lipophilic polyphenolic compound extracted from turmeric root. It has broad-spectrum antitumor activity due to its anticancer properties. Its anti-GC effects are mainly realized through the following mechanisms:

1. Inhibition of proliferation and induction of apoptosis: Studies have shown that Cur induces apoptosis and inhibits the proliferation, migration, and invasion of GC cells in a dose- and time-dependent manner. It induces apoptosis and inhibits proliferation by upregulating long non-coding RNA (lncRNA) AC022424.2, inhibiting the PI3K/AKT/mTOR signaling pathway, and activating the NF-κB signaling pathway (73).
2. Reversal of chemoresistance and enhancement of therapeutic efficacy: It has been shown that Cur can overcome GC chemoresistance by inhibiting the activation of the cancer-associated fibroblast-mediated JAK/STAT3 signaling pathway (74). In addition, the combination of Cur and metformin can enhance the cytotoxic effects of chemotherapeutic agents on GC cells (75).
3. Nanodelivery system to optimize efficacy: To address the low water solubility and bioavailability of Cur, researchers developed chitosan nanocapsules and cationic peptide-modified polyhydroxyalkanoate (PHA) nanospheres (Cur@PHBX-PR/FUdR15S). Nanocapsules were found to be safer and more effective in inhibiting the growth of *H. pylori* than nanoemulsions (76). The cationic peptide-coated PHA nanosphere Cur@PHBX-PR/FUdR15S enhanced the activity of apoptosis-associated proteins and FUdR-sensitive GC cells by synergistically loading chemotherapeutic agents such as FUdR15S (8).

In summary, Cur regulates the malignant biological behavior of GC through multi-targets, and its combination with nanotechnology provides a new strategy to address pharmacokinetic deficiencies with potential for clinical translation. However, the mechanisms of action are diverse and the primary target sites have not yet been fully identified. Furthermore, the long-term safety, the batch-to-batch consistency, and the clinical manufacturability of the relevant nanomedicines require further validation.

### 3.3 Terpenoids

#### 3.3.1 Cantharidin

Cantharidin (CTD), a terpenoid isolated from blister beetles (The mummified remains of a spotted moth and Spotted Moth of the family Mothidae), is now widely used in cancer therapy. Its mechanisms of action in GC therapy are mainly characterized by the following:

1. Inhibition of GC cell proliferation and metastasis and induction of apoptosis: Song et al. further demonstrated that CTD inhibited the PI3K/AKT signaling pathway through the regulation of CCAT1, thereby reducing GC cell invasion and metastasis (77). In addition, CTD combined with 5-FU chemotherapy synergistically reduced GC cell activity and increased the expression of phosphorylated p38 MAPK and phosphorylated JNK associated with C-MYC and p53 to induce apoptosis (78).
2. Inhibition of tumor angiogenesis and modulation of cell adhesion: Zhang et al. found that norcantharidin (NCTD) significantly inhibited tumor angiogenesis by blocking the VEGFR2/MEK/ERK signaling pathway (79). Notably, a comprehensive analysis using an antitumor proprietary medicine database, network pharmacology, and transcriptomics showed that NCTD could treat GC by interfering with the PI3K/AKT, NF-κB, and other signaling pathways, as well as the EMT-related pathway, to modulate biological processes such as cell adhesion, migration, and inflammatory responses (80).

Available evidence suggests that CTDs, as terpenoids of natural origin, show significant promise for application in GC therapy due to their multi-targeted antitumor properties.

#### 3.3.2 Dihydroartemisinin

DHA is a sesquiterpene lactone active compound extracted from *Artemisia annua*, a TCM plant. DHA, as a key active derivative of artemisinin, possesses anti-inflammatory, antioxidant, and anti-fibrotic properties. It is not only widely used in the treatment of malaria but has also been shown to have anti-GC efficacy. Existing studies have shown that its mechanisms of action are mainly reflected in the following aspects:

1. Inhibition of GC angiogenesis: Anti-angiogenic therapy is a salvage treatment for patients with advanced GC; however, its efficacy is not satisfactory because the blood supply of GC tissues involves endothelium-dependent vessels and angiogenic mimicry (vasculogenic mimicry, VM) independent of endothelial cells. DHA can inhibit the expression of FGF2 and the formation of VM in GC *in vivo* and *in vitro*. Specifically, as follows, DHA effectively inhibits FGF2 expression, reduces FGFR1 receptor activation, inhibits intracellular signaling pathway activation, such as the Ras/Raf/MEKs/MAPK and PI3K/AKT/mTOR signaling pathways, and represses nuclear transcription, such as MMP-9, Twist1, vimentin, MMP-2, and VE-cadherin, as well as VM formation (81).
2. Inhibition of GC cell proliferation and metastasis, induction of programmed death, and reversal of chemoresistance: It can also inhibit the proliferation and migration of GC cells by inhibiting tankyrases, which is related to the inactivation of the Wnt/β-catenin pathway and the EMT process (82). Another study showed that DHA effectively inhibited the proliferation, invasion, and migration of cisplatin (diamminedichloroplatinum, DDP)-resistant and GC cells and induced apoptosis. The mechanisms involved enhancement of autophagy through the inhibition of the PI3K/AKT/mTOR pathway, apoptosis induced by caspase-dependent and mitochondrial pathways, and enhancement of cisplatin sensitivity through P-gp inhibition (83). DHA in combination with DDP synergistically inhibited GC cell proliferation, invasion, and migration and induced GC cell ferroptosis by inhibiting GPX4 *in vivo* and *in vitro* (7).
3. Nanodelivery system to optimize efficacy: DHA has poor water solubility, which affects its oral efficacy. Forming a ternary system with hydroxypropyl-β-cyclodextrin and lecithin can simultaneously enhance its solubility and stability, providing a simple and effective formulation strategy for improving its oral bioavailability (84). However, for the clinical translation of this approach in the treatment of GC, further validation in animal models is required to demonstrate its pharmacokinetic and pharmacodynamic advantages over free DHA, as well as its long-term storage stability, feasibility of large-scale production, and long-term safety.

Existing evidence suggests that DHA exhibits significant medical value in GC treatment through multidimensional mechanisms of action, such as modulation of the proliferation–apoptosis imbalance, remodeling of the angiogenic microenvironment, and reversal of chemotherapy resistance, and has significant development potential as a naturally sourced multi-target inhibitor.

#### 3.3.3 Oridonin

Oridonin (ORI) is a diterpenoid isolated from the dried aboveground parts of the plant *Rabdosia rubescens* (Hemsl.) of the family Lipaceae, which has significant anti-GC effects. Its mechanisms of action in GC therapy are mainly characterized by the following:

1. Inhibition of GC cell proliferation: ORI inhibits GC cell proliferation by regulating the TNF-α/AR/TGF-β signaling pathway (85).
2. Induction of ferroptosis: The ORI derivative Jiyuan oridonin A2 (JDA2) induced ferroptosis in GC cells through the downregulation of GPX4, concomitant autophagy pathway-mediated accumulation of ferrous iron (Fe2^+^), and the generation of large amounts of lipid ROS through the reaction of Fe2^+^ with ROS. The degree of GPX4 downregulation serves as a predictive marker of JDA2 sensitivity, making it a promising GC therapeutic agent (86).
3. Nanodelivery systems optimize efficacy:
  - Redox-responsive delivery: To overcome the problems of the poor solubility, low bioavailability, and rapid plasma clearance of ORI, researchers covalently attached ORI to poly(ethylene glycol)-block-poly(l-lysine) (PEG-b-PLL) via disulfide bonds and synthesized a redox-sensitive ORI polymer pre-drug formulation, which can be self-assembled in an aqueous solution into nanomicellar carriers (P-ss-ORI). The micelles could rapidly and completely release ORI under glutathione (GSH)-rich and low-pH conditions, effectively inhibiting GC growth (87).
  - ROS-responsive co-delivery: Another study designed nanoparticles (NPs) for the co-delivery of ORI and DHA as combination therapy for GC. The NPs synthesize ROS-responsive ORI and DHA polymeric precursors by coupling ORI or DHA with PEG-b-PLL via a ROS-sensitive linker thioketal (TK) that self-assembles to form NPs (OD-M) in water. Upon internalization by GC cells, OD-M releases ORI and DHA under high-ROS conditions in cancer cells. The released ORI reacts with GSH to induce GSH depletion, and DHA exacerbates the intracellular ROS levels, ultimately leading to the ferroptosis of GC cells (88).

Available evidence suggests that ORI and its derivatives exert anti-GC effects through the modulation of the key signaling pathways and the induction of ferroptosis. The combination of advanced nanodelivery technology significantly enhances their bioavailability and targeting, in particular their unique mechanism in inducing ferroptosis, which makes them highly promising anti-GC candidates of natural origin.

#### 3.3.4 Pachymic acid

Pachymic acid (PA) is a naturally occurring triterpenoid in the TCM *Poria cocos* (PC) with anti-inflammatory, antioxidant, and antitumor properties. Studies have shown that PA inhibits GC progression through a multi-target regulatory mechanism.

Network pharmacology has shown that PA inhibits GC cell proliferation by regulating key targets such as MAPK1 and PIK3R1, with the inactivation of the PIK3R1-mediated PI3K/AKT signaling pathway as the core mechanism. It was confirmed that PA blocked the PI3K/AKT pathway, induced cell cycle arrest, and promoted apoptosis (37). In another study, PA was found to inhibit GC development by interacting with PDGFRB, which counteracted the promoting effects of PDGFRB overexpression on the proliferation, migration, and invasion of GC cells; inhibited the activation of the PI3K/AKT signaling pathway; and induced ferroptosis in GC cells (38).

Therefore, the use of PA to regulate PI3K/AKT signaling and induce apoptosis or ferroptosis in GC cells provides a new avenue for future therapeutic GC drug research.

#### 3.3.5 Celastrol (thunder god vine)

Celastrol is a triterpenoid natural active compound extracted from the TCM thunder god vine, which has significant anti-GC activity. Its mechanisms of action in GC treatment are mainly reflected in the following aspects:

1. Inhibition of GC cell proliferation and progression: Wu et al. found that celastrol inhibited tumor growth and increased ROS in a STAT3-dependent manner in GC cell lines. At the molecular level, it downregulated interleukin 6 (IL-6) levels by inhibiting the activation and transcriptional activity of STAT3 and suppressed the JAK2/STAT3 signaling pathway, thus inhibiting GC progression (89). Wei et al. found that celastrol could inhibit GC progression by blocking the PI3K/AKT pathway through inhibition of the FOXA1/claudin-4 axis (90).
2. Induction of apoptosis and reversal of drug resistance in GC cells: It triggers apoptosis by inducing the degradation of the oncogenic inhibitor cancerous inhibitor of PP2A (CIP2A) and activating the PP2A/GSK3β axis, leading to decreased myeloid cell leukemia-1 (MCL-1) expression (91). In addition, celastrol inhibits the proliferation of cisplatin-resistant GC cells, induces apoptosis, and reduces the expression of drug-resistant genes, an effect associated with the inhibition of the expression of the proteins related to the mTOR signaling pathway (92).
3. Inhibition of GC angiogenesis and metastasis: The most promising celastrol derivative compounds can inhibit tumor angiogenesis and metastasis by blocking GC cells in the G2 phase, inhibiting the expression of the vascular endothelial growth factor, and downregulating the metastasis-related protein MMP-9 (93).

Available evidence suggests that celastrol exerts multiple anti-GC effects, including inhibition of proliferation, induction of apoptosis, reversal of drug resistance, and anti-angiogenesis and metastasis by regulating multiple signaling pathways and key targets, demonstrating its value as a multi-targeted natural antitumor drug in GC treatment.

### 3.4 Carotenoids

#### 3.4.1 Crocin

Crocin is a naturally occurring carotenoid from saffron with antioxidant, anti-inflammatory, and antitumor activities. It inhibits GC progression through multiple mechanisms: crocin promotes the translocation of Nrf2 from the nucleus to the cytoplasm, leading to its inactivation and the inhibition of the transcription of the oncogene GGTLC2, which downregulates the expression of this oncogene. Furthermore, crocin may inhibit the onset and progression of GC by promoting ferroptosis and apoptosis in GC cells through modulation of the Nrf2/GGTLC2 pathway (94). In addition, crocin can inhibit GC cell proliferation by suppressing the expression of TPM4, a key regulator of cell proliferation (95). It can also combine with DDP to inhibit GC proliferation, apoptosis, and EMT through the FGFR3/MAPK/ERK pathway (96). In conclusion, crocin can treat GC by inducing GC cell death, and it shows great potential in the treatment of GC.

### 3.5 Others

In addition to the above compounds, a variety of natural products have therapeutic effects on GC.

Berberine (Ber) is an alkaloid isolated from *Berberis vulgaris* and other *Berberis* species. It inhibits the IL-6/JAK2/STAT3 signaling pathway *in vitro* and *in vivo*, in turn inhibiting GC proliferation (97). A novel chitosan/pectin NP containing Ber (NPs-Ber) has also been formulated to address the delivery issue in GC cells. The level of 5-methylated cytosine (5-mC) was significantly higher in cells treated with NPs-Ber than in cells treated with unloaded NPs or free Ber, which enhanced its cytotoxicity and epigenetic effects on GC (98).

Andrographolide is a diterpenoid lactone from *Andrographis paniculata*, and its derivative inhibits the ERK/c-Fos/Jun pathway and potently and selectively suppresses GC cell proliferation and migration (99).

Isoliquiritigenin (ISL) is a natural flavonoid derived from the roots of licorice (*Glycyrrhiza uralensis*, commonly referred to as the licorice plant), which has been shown to inhibit GLUT4-mediated glucose uptake and to induce PDHK1/PGC-1α-mediated energy metabolism collapse by downregulating the protein expression of c-Myc and HIF-1α in GCs, which inhibits GC growth (100).

Ginkgolide B (GGB) is a diterpenoid lactone derived from *Ginkgo biloba*, which exerts its anti-GC effects in a multifaceted manner by inhibiting the PI3K/AKT/m-TOR pathway dose-dependently, inhibiting proliferation and promoting apoptosis and pyroptosis (101).

In addition, there are other TCM ingredients that not only have anti-GC effects but also improve the sensitivity or overcome the resistance to anticancer drugs. Paeonol, a phenolic substance isolated from *Paeonia suffruticosa* Andrews, can inhibit the proliferation, migration, invasion, and glycolysis of apatinib-resistant GC cells and promote apoptosis by regulating the LINC00665/miR-665/MAPK1 axis, representing a potential drug for reversing drug resistance in GC cells (102).

**Figure 2:**
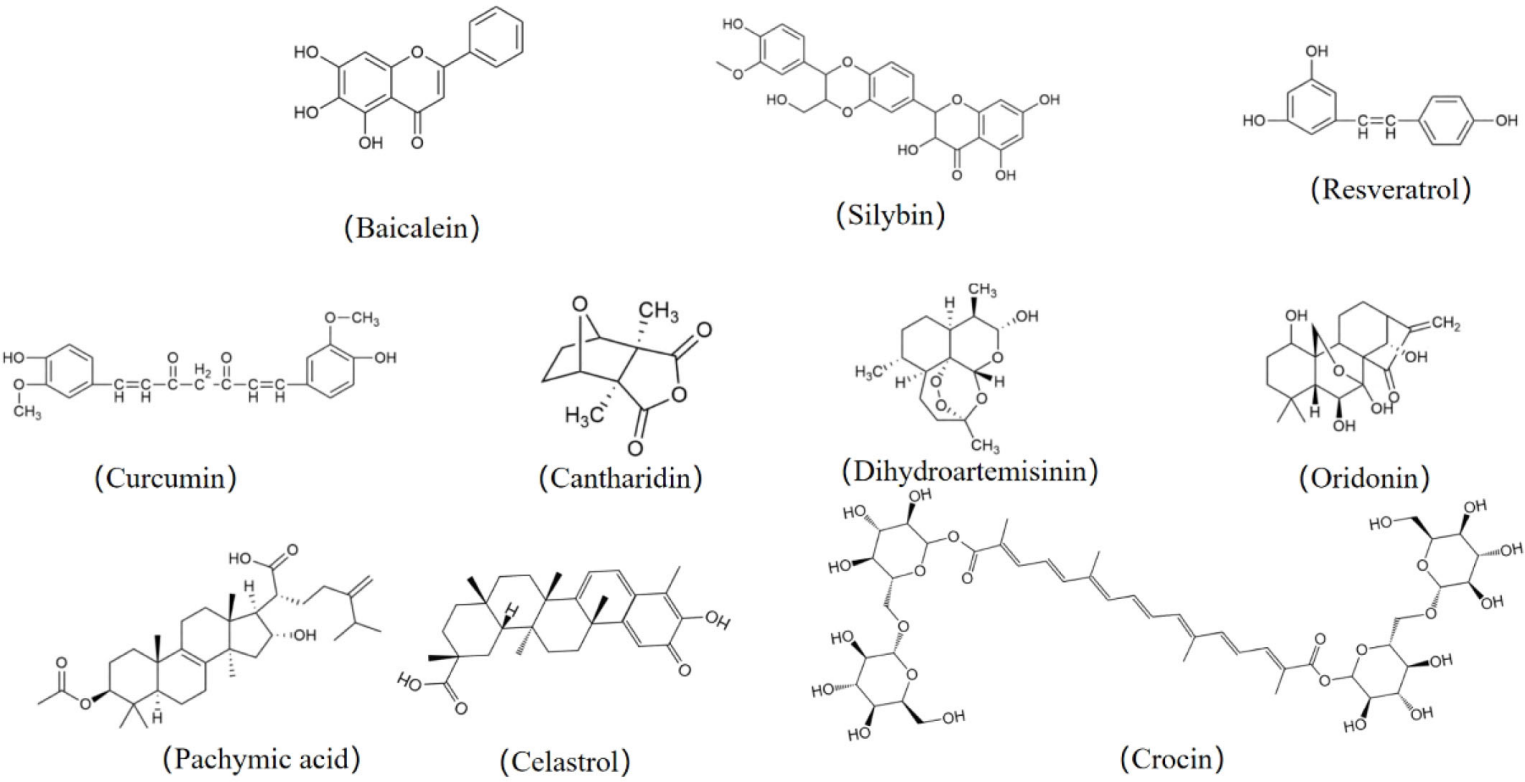
Structural formulas of the various Chinese medicine monomers (CMMs).

**Figure 3:**
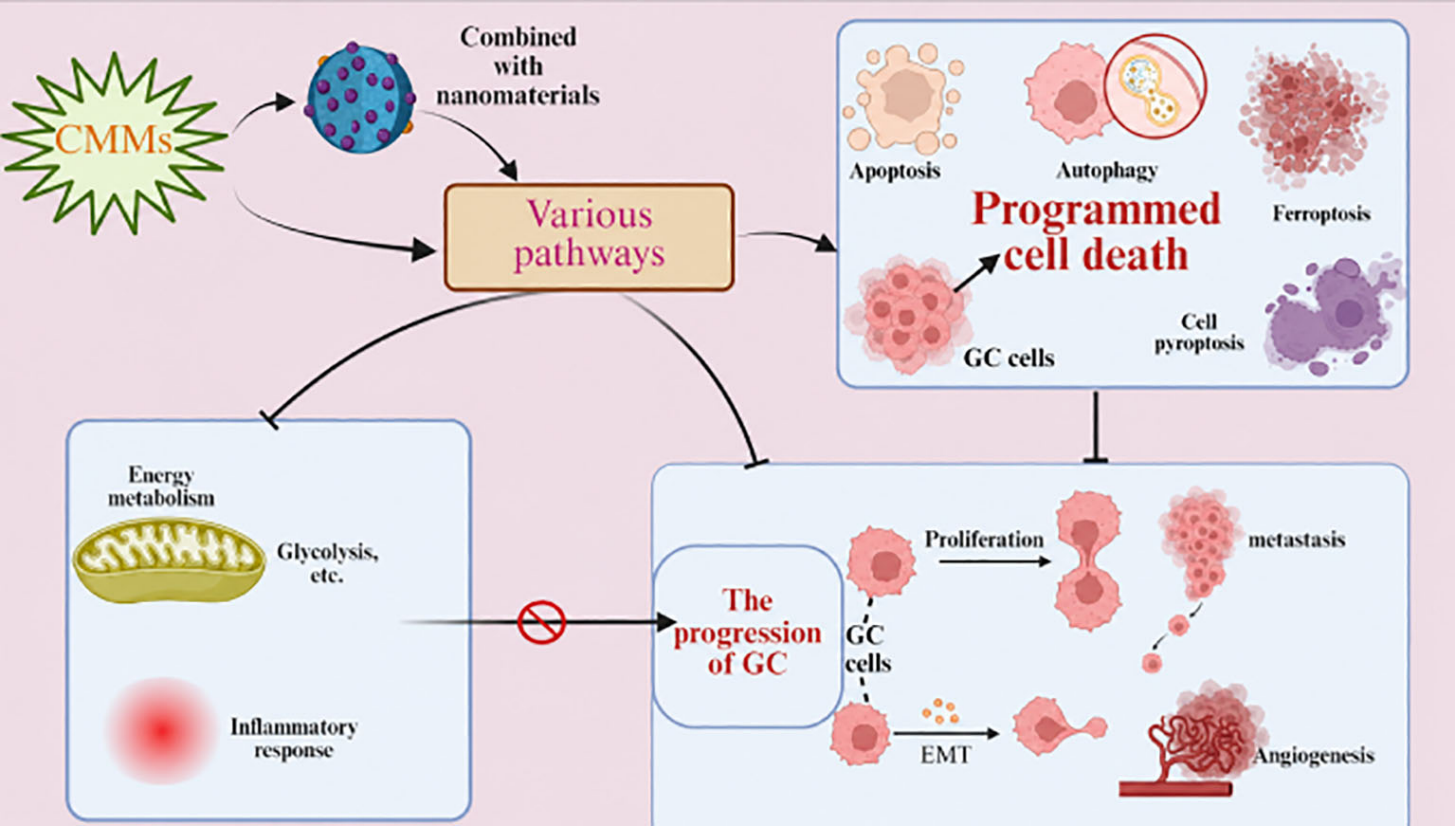
Mechanisms of gastric cancer (GC) treatment by Chinese medicine monomers (CMMs).

In conclusion, these CMMs show great potential for anti-GC in directly inhibiting GC progression by targeting multiple key signaling pathways, enhancing therapeutic effects in combination with nanomaterials, and overcoming drug resistance. **Table 2** displays the therapeutic efficacy of CMMs in GC. See **Figure 2** for the structural formula and **Figure 3** for the mechanisms of action of CMMs in the treatment of GC.

**Table 2:** Therapeutic effects of Chinese medicine monomers (CMMs) on gastric cancer (GC).

Monomer	Related signaling pathways/targets	Effects	Related traditional Chinese medicine	Reference
Baicalin	Bax, Bcl-2, caspase-3, and caspase-9 NF-κB–NLRP3	Inhibit the proliferation of GC cells Induce apoptosis, pyroptosis, and ferroptosis Enhance chemotherapy sensitivity	*Scutellaria baicalensis*	(5, 6, 61, 62)
Silybin	miR-181a and miR-34a TGFB/SMAD3/β-catenin NF-κB/STAT3 COX-2 and iNOS	Inhibit the proliferation of GC cells Induce cell apoptosis Alleviate the inflammatory response induced by *Helicobacter pylori* infection	*Silybum marianum*	(63–65)
Resveratrol	NF-κB MALAT1/miR-383-5p/ DDIT4 FOS and MMP-9 SIRT3	Inhibit the proliferation, invasion, and metastasis of GC cells Induce cell apoptosis and autophagy Enhance chemotherapy sensitivity Optimize the therapeutic effect by combining the nanodelivery system	*Veratrum grandiflorum*	(9, 67–72)
Curcumin	lncRNA AC022424.2 NF-κB and PI3K/AKT/mTOR JAK/STAT3	Inhibit the proliferation of GC cells Induce cell apoptosis Reverse chemotherapy resistance Enhance the therapeutic effect Optimize the therapeutic effect by combining the nanodelivery system	Turmeric root	(8, 73–76)
Cantharidin	CCAT1 p-p38 and p-JNK VEGFR2/MEK/ERK PI3K/AKT NF-κB	Inhibit the proliferation and metastasis of GC cells Induce cell apoptosis Inhibit tumor angiogenesis Regulate cell adhesion	Blister beetle	(77–80)
Dihydroartemisinin	Ras/Raf/MEKs/MAPK PI3K/AKT/mTOR Wnt/β-catenin, tankyrases MMP-9, Twist1, vimentin, MMP-2, VE-cadherin, P-gp, and caspase	Inhibit the proliferation and metastasis of GC cells Enhance autophagy Induce apoptosis and ferroptosis Reverse chemotherapy resistance Inhibit GC angiogenesis	*Artemisia annua*	(7, 81–84)
Oridonin	TNF-α/AR/TGF-β GPX4	Inhibit the proliferation of GC cells Induce ferroptosis Optimize the therapeutic effect by combining the nanodelivery system	*Rabdosia rubescens* (Hemsl.)	(85–88)
Pachymic acid	MAPK1 PIK3R1, PI3K/AKT PDGFRB	Inhibit the proliferation of GC cells Induce apoptosis and ferroptosis	*Poria cocos*	(37, 38)
Celastrol	JAK2/STAT3, PI3K/AKT FOXA1/claudin-4 PP2A-GSK3β, CIP2A, MCL-1, mTOR, and MMP-9	Inhibit the proliferation of GC cells Induce apoptosis of GC cells Reverse drug resistance Inhibit the generation and metastasis of GC vessels	Thunder god vine	(89–93)
Crocin	Nrf2/GGTLC2 Tropomyosin alpha 4 FGFR3/MAPK/ERK	Inhibit the proliferation of GC cells Induce apoptosis and ferroptosis of GC cells	*Saffron crocus*	(94–96)
Berberine	IL-6/JAK2/STAT3	Inhibit the proliferation of GC cells Optimize the therapeutic effect by combining the nanodelivery system	*Coptis chinensis*	(97, 98)
Andrographolide	ERK/c-Fos/Jun	Inhibit the proliferation and metastasis of GC cells	Green chiretta	(99)
Isoliquiritigenin	GLUT4 c-Myc and HIF-1α PDHK1/PGC-1α	Inhibit the proliferation of GC cells	*Glycyrrhiza uralensis*	(100)
Ginkgolide B	PI3K/AKT/m-TOR	Inhibit the proliferation of GC cells Induce apoptosis and pyroptosis	*Ginkgo* leaf	(101)
Paeonol	LINC00665/miR-665/MAPK1	Inhibit the proliferation, migration, invasion, and glycolysis of GC cells Induce cell apoptosis	Paeonia suffruticosa Andrews	(102)

## 4 Discussion

CMMs have garnered considerable attention in oncology for their multi-constituent synergism, multi-targeted actions, and favorable toxicity profiles. They can regulate the biological behaviors of tumor cells, such as proliferation, survival, autophagy, invasion, and metastasis, through multiple pathways. This review systematically elucidates the important GC-related signaling pathways, as well as the anti-GC therapeutic effects and the molecular mechanisms exerted by representative CMMs, through interfering with the relevant signaling pathways. The isolation of bioactive constituents from CMMs or their structural optimization to develop potent antitumor agents represents a critical strategy in contemporary drug discovery. The CMMs discussed in this paper are expected to provide new avenues for research and clinical translation in the prevention and treatment of GC; however, they require further validation through high-quality patient studies and prospective clinical trials.

### 4.1 Mechanism of CMMs against GC

Extensive preclinical research has demonstrated that CMMs can inhibit the onset and progression of GC through multiple mechanisms and pathways. The main mechanisms are as follows:

1. Inhibition of GC cell proliferation, invasion, and metastasis. For example, CTD can inhibit GC proliferation and reduce GC cell invasion and metastasis by inhibiting the PI3K/AKT signaling pathway.
2. Induction of apoptosis and autophagy in GC cells. For example, Res sensitized GC cells to 5-FU by activating the apoptosis and autophagy pathways, enhancing the efficacy of 5-FU in GC treatment.
3. Induction of GC cell pyroptosis and ferroptosis. For example, DHA can induce ferroptosis in GC cells by inhibiting GPX4 *in vivo* and *in vitro*. Baicalin induces GC pyroptosis via the NF-κB–NLRP3 signaling pathway.
4. Inhibition of tumor angiogenesis. For example, CTD, DHA, and celastrol can regulate relevant signaling pathways to reduce tumor angiogenesis.
5. Enhancement of chemotherapy sensitivity or overcoming chemotherapy resistance. For example, Cur can overcome chemoresistance by inhibiting cancer-associated fibroblasts from activating the JAK/STAT3 signaling pathway in GCs, while 4-BR can inhibit GC cell stemness and increase chemosensitivity through the SIRT3/c-Jun N-terminal kinase pathway.
6. Combination of nanomaterials (using nanodelivery systems) to enhance therapeutic efficacy. The combination of TCM with nanomaterials has shown potential for addressing pharmacokinetic issues such as low solubility, low bioavailability, and rapid plasma clearance. Nanodelivery systems can circumvent the issues of poor selectivity and damage to healthy cells associated with traditional cancer therapies, thereby ensuring that drugs reliably reach tumor tissues and reducing the side effects caused by off-target clinical applications. For example, Res, Cur, and ORI have utilized the advantages of nanomaterials to exert anti-GC effects.

### 4.2 Current status of clinical evidence

The current body of evidence is primarily derived from preclinical studies, while prospective clinical trials directly evaluating the efficacy and safety of CMMs in patients with GC remain relatively limited. Existing clinical evidence focuses more on the differences in the expression of pathway molecules in patient tissues, their prognostic relevance, and their potential value as biomarkers: examples include the association between abnormal expression of Notch ligands and overall survival, as well as the predictive value of *NOTCH4* mutations in the response to immunotherapy. While these findings support the importance of the relevant signaling pathways in GC at the patient level, they do not equate to clinical validation of CMMs. Consequently, the anti-GC effects summarized in this paper should primarily be defined as therapeutic potential supported by preclinical evidence rather than established clinical efficacy.

### 4.3 Limitations and future directions

Although CMMs have demonstrated potential for multi-targeted therapy in GC research, their clinical translation still faces multiple limitations. Firstly, CMMs have a complex chemical composition: individual herbal ingredients often contain multiple active components, and the interactions between the various components in compound formulations remain unclear, posing a challenge to the comprehensive elucidation of their pharmacological mechanisms. Secondly, pharmacokinetic barriers represent a key bottleneck: the majority of CMMs have poor water solubility, low oral bioavailability, and are rapidly metabolized *in vivo*, making it difficult to achieve effective therapeutic concentrations in tumor tissues. Thirdly, the variability of TCM formulations cannot be overlooked: factors such as the origin of the herbal materials, the harvesting season, and the extraction processes can all lead to variations in the content and purity of the same constituent across different batches, thereby affecting quality control and the stability of therapeutic efficacy. Lastly, although CMMs are generally considered to have a good safety profile, their potential organ toxicity and off-target effects on normal tissues, particularly with increased doses or long-term use, still require rigorous evaluation through standardized preclinical and clinical studies. Furthermore, as the majority of the existing evidence is based on animal experiments or *in vitro* studies, the lack of prospective clinical trial data significantly weakens the persuasiveness of the conclusions.

In light of these limitations, future research should focus on the following areas:

1. Overcome pharmacokinetic bottlenecks: utilize nanodelivery systems to enhance the solubility, bioavailability, and tumor-targeting of CMMs and explore pro-drug modification strategies.
2. Establish quality control standards: standardize the homogeneity of CMM raw materials and formulations using methods such as fingerprinting and quantitative analysis to ensure the reproducibility of the experimental and clinical research results.
3. Systematically evaluate toxicity profiles: conduct standardized *in vivo* toxicological studies and long-term follow-ups to clarify the safety margin, dose-limiting toxicity, and potential adverse reactions associated with the combination of CMMs and chemotherapeutic agents.
4. Deepen research into molecular mechanisms: utilize techniques such as chemical biology and single-cell sequencing to precisely identify the direct targets of CMMs and the pathways associated with drug resistance.
5. Advance high-quality clinical research: design randomized controlled trials or real-world studies to validate the efficacy and safety of CMMs used alone or in combination with chemotherapy/immunotherapy, thereby providing high-level evidence for clinical translation.

## 5 Conclusion

This review systematically summarizes the regulatory role of CMMs in the key signaling pathways of GC, supporting their clinical therapeutic potential in enhancing sensitivity to chemotherapy and in integration with nanotechnology. To fully realize the clinical potential of CMMs, future research should prioritize the identification of precise molecular targets and validate the synergistic effects of combination formulations through rigorously designed clinical trials. This will provide a theoretical foundation and practical guidance for the development of safer and more effective treatment strategies for GC. Continued in-depth basic research and clinical translation efforts will help advance the standardization of CMMs in the field of GC treatment.

## Glossary

APC: adenomatous polyposis coli
AKT: protein kinase B
Bcl-2: B-cell lymphoma-2
BAD: BCL2-associated agonist of cell death
BaX: BCL2-associated X protein
c-Fos: cellular Finkel–Biskis–Jinkins murine osteosarcoma viral oncogene homolog
c-Jun: cellular Jun proto-oncogene
C-MYC: myelocytomatosis oncogene
COX-2: cyclooxygenase-2
CCAT1: colon cancer associated transcript 1
DDIT4: DNA damage inducible transcript 4
DLL1/3/4: delta-like protein1/3/4
ERK: extracellular signal-regulated kinase
EMT: epithelial–mesenchymal transition
FGF2: fibroblast growth factor 2
FGFR1/3: fibroblast growth factor receptor 1/3
FOS: FBJ murine osteosarcoma viral oncogene homolog
FOXA1: forkhead box A1
Fas: Fas receptor
FasL: Fas ligand
FUdR: 5-fluorodeoxyuridine
FADD: Fas-associated death domain
GLI1: glioma-associated oncogene homolog 1
GGTLC2: gamma-glutamyltransferase light chain 2
GSH: glutathione
GSK3β: glycogen synthase kinase 3β
GLUT4: glucose transporter type 4
HGC-27: human gastric cancer cell line-27
HIF-1α: hypoxia-inducible factor 1-α
iNOS: inducible nitric oxide synthase
IL-6: interleukin 6
JAK: Janus kinase
JAG1/2: Jagged1/2
JNK: c-Jun N-terminal kinase
LRP: low-density lipoprotein receptor-associated protein
mTOR: mammalian target of rapamycin
MEK: mitogen-activated protein kinase
MMP2/9: matrix metallopeptidase 2/9
MALAT1: metastasis associated lung adenocarcinoma transcript 1
NF-κB: nuclear factor-κB
NLRP3: NOD-like receptor 3
Nrf2: nuclear factor erythroid 2-related factor 2
PA: pachymic acid
PGC-1α: peroxisome proliferator-activated receptor gamma coactivator 1-alpha
PDHK1: pyruvate dehydrogenase kinase 1
PDGFRB: platelet-derived growth factor receptor β
PP2A: protein phosphatase 2A
P-gp: P-glycoprotein
PTCH1/2: patched homolog1/2
PTEN: phosphatase and tensin homolog
PHLDB: Pleckstrin homology-like domain family B
PI3K: phosphatidylinositol-3-kinase
RAS: rat sarcoma viral oncogene homolog
RAF: rapidly accelerated fibrosarcoma
STAT: signal transducer and activator of transcription
SIRT3: silent information regulator 3
SMAD3: small mothers against decapentaplegic 3
TNF-α: tumor necrosis factor-α
Twist1: Twist family BHLH transcription factor 1
TCF4: T-cell factor 4
TYK-II: tyrosine kinase 2
TGF-β: transforming growth factor β
VE-cadherin: vascular endothelial-cadherin
VEGFR2: vascular endothelial growth factor receptor 2
Wnt: Wingless/Int-1
YWHAZ: tyrosine 3-monooxygenase/tryptophan 5-monooxygenase activation protein zeta
ZIF-90: zeolitic imidazolate framework-90.
